# H_2_S Removal from Groundwater by Chemical Free Advanced Oxidation Process Using UV-C/VUV Radiation

**DOI:** 10.3390/molecules26134016

**Published:** 2021-06-30

**Authors:** Yael Gilboa, Yuval Alfiya, Sara Sabach, Eran Friedler, Yael Dubowski

**Affiliations:** Faculty of Civil and Environmental Engineering, Technion-Israel Institute of Technology, Haifa 3200, Israel; ygilboa@technion.ac.il (Y.G.); alfiya@cv.technion.ac.il (Y.A.); sarass@cv.technion.ac.il (S.S.); eranf@technion.ac.il (E.F.)

**Keywords:** hydrogen sulfide, advanced oxidation process (AOP), vacuum-UV (VUV), photo-oxidation, groundwater, water treatment

## Abstract

Sulfide species may be present in groundwater due to natural processes or due to anthropogenic activity. H_2_S contamination poses odor nuisance and may also lead to adverse health effects. Advanced oxidation processes (AOPs) are considered promising treatments for hydrogen-sulfide removal from water, but conventional AOPs usually require continuous chemical dosing, as well as post-treatment, when solid catalysts are applied. Vacuum-UV (VUV) radiation can generate ·OH in situ via water photolysis, initiating chemical-free AOP. The present study investigated the applicability of VUV-based AOP for removal of H_2_S both in synthetic solutions and in real groundwater, comparing combined UV-C/VUV and UV-C only radiation in a continuous-flow reactor. In deionized water, H_2_S degradation was much faster under the combined radiation, dominated by indirect photolysis, and indicated the formation of sulfite intermediates that convert to sulfate at high radiation doses. Sulfide was efficiently removed from natural groundwater by the two examined lamps, with no clear preference between them. However, in anoxic conditions, common in sulfide-containing groundwater, a small advantage for the combined lamp was observed. These results demonstrate the potential of utilizing VUV-based AOP for treating H_2_S contamination in groundwater as a chemical-free treatment, which can be especially attractive to remote small treatment facilities.

## 1. Introduction

Groundwater often serves as an important water source, especially in arid and semiarid regions. Industrial activity, as well as natural processes, may result in contamination of this water by various pollutants, including sulfide compounds. In low-oxygen aquatic environments, as often found in groundwater in confined aquifers, hydrogen sulfide (H_2_S) and bisulfide (HS^−^) may accumulate following anaerobic reduction of sulfate by bacteria [1]. H_2_S is a hazardous and fragrant contaminant that poses odor nuisances already at concentrations above 0.05 mg/L, and at concentrations above 1 mg/L, it may also lead to adverse health effects [2,3,4]. Furthermore, the presence of H_2_S in drinking water may also affect its taste as well as accelerate corrosion processes in water distribution and treatment systems. In an aqueous system, H_2_S dissociates to HS^−^ and S^2−^ with pKa’s of 7.05 and 12.9, respectively. Hence, under the common pH range of groundwater (6–8.5), H_2_S and HS^−^ are likely to be the dominant species.

Various treatment approaches have been established for H_2_S removal from water, including ventilation, adsorption on activated carbon and on ion exchange resins, chemical precipitation (by metals such as Fe^3+^, Cu^2+^, and Zn^2+^), biological oxidation, and chemical oxidation (mainly by O_3_, Cl_2_, KMnO_4_, or H_2_O_2_; e.g., [5,6,7,8,9]). While ventilation and sorption processes only transfer untreated pollutants to different media (hence requiring further treatment), the other techniques actually transform H_2_S to other compounds. Nevertheless, these latter techniques exhibit major drawbacks that pose additional complexation and costs to the treatment process; chemical oxidation and precipitation require the controlled addition of chemicals as well as the removal of solids if formed, whereas biological oxidation of hydrogen-sulfide species is often slow and very sensitive to operating conditions.

Following these disadvantages with the aim of efficient oxidation of sulfides, there has been growing attention in applying advanced oxidation processes (AOPs) using ·OH radicals for sulfide removal from water systems ([2] and references therein). Hydroxyl radicals are highly reactive toward all hydrogen-sulfide species, with second-order reaction rate coefficients of 1.5 × 10^10^, ~1 × 10^10^, and 4 × 10^9^ M^−1^S^−1^ for H_2_S, HS^−^, and S^2−^, respectively [5]. In most AOPs ·OH generation is conducted via UV-C irradiation (254 nm) with the addition of H_2_O_2_, TiO_2_, or O_3_ [2,10,11]. Recently, Tzvi and Paz [12] investigated the direct role of UV-C radiation in the oxidation of H_2_S as well as proposed a new mechanism for H_2_S oxidation in low-turbidity well-water, based on the absorption of UV-C light by HS^−^ in an oxygen-containing environment. While AOPs, as well as direct photolysis in oxygen-augmented solution, have proven efficient for sulfide oxidation to harmless sulfate, they still require chemicals addition. This disadvantage is especially important for sulfide enriched groundwater, which is usually anoxic and needs to be treated in remote sites.

A promising alternative that overcomes this limitation is the application of VUV radiation (λ < 200 nm). Under such radiation, water photolysis generates ·OH and other radicals in situ without chemical additions (Reactions 1–2, [13]), giving the process a potential economic and operational advantage.

H_2_O + hν (<190 nm) → ·OH + H·             Φ(·OH ) = 0.33 (1)

H_2_O + hv (<190 nm) → e^−^ _(aq)_ + ·OH + H^+^   Φ(e^−^ _(aq)_) = 0.045 (2)

The direct formation of hydroxyl radicals makes VUV radiation among the most advanced oxidation processes [14]. In recent years, there is accumulating evidence for the potential of a VUV-based AOP for removal of persistent pollutants both in deionized water (e.g., [15,16]) as well as in more realistic water matrices (e.g., [17,18,19,20,21]). Like all AOP processes, a successful VUV-based AOP needs to minimize the formation of undesired intermediates and overcome interferences from other compounds that may compete for the hydroxyl radicals formed (e.g., carbonate, bicarbonate, chloride, and natural organic matter) or act as inner UV filters (e.g., nitrates and NOM) (e.g., [21,22]). An additional challenge for this VUV-based AOP is the limited penetration depth of VUV radiation in water (approximately 11 mm at 185 nm; [23]), which requires special attention in reactor designing and probably makes this process more attractive to decentralized and small-scale water treatment facilities that treat smaller volumes of water.

While there is accumulating evidence for the potential of a VUV-based AOP for treating various pollutants in water systems, only a few studies have investigated H_2_S removal by VUV radiation, and even those were conducted with gaseous H_2_S [24,25,26,27]. Li et al. [26], who investigated H_2_S_(g)_ degradation under 365, 254, and 185 nm wavelengths, showed a considerably higher reduction in its concentration when irradiated by 185 nm light (97% removal compared to 33% and 39% removal under 365 nm and 254 nm light). These authors further reported a much faster H_2_S removal rate when humidity was increased in the presence of the VUV radiation (185 nm). Enhanced H_2_S degradation under VUV irradiation may result from direct photolysis by these short wavelengths and/or due to oxidation by the ·OH radicals (and H_2_O_2_) generated in the solution following water homolysis. The above-mentioned findings suggest that oxidation by ·OH radicals, which form in the gaseous phase by reaction between singlet atomic oxygen and water vapor (Reactions (3)–(6), [28]), plays a dominant role in H_2_S oxidation under VUV radiation.

O_2(g)_ + hν (λ < 240 nm) **→** 2O (3)

O_2_ + O **→** O_3_ (4)

(5) $$O_{3}+h\nu \;(\lambda \;<\;320\;\mathrm{nm})\;↔\;O+O_{2}$$

H_2_O(g) + O (^1^D) **→** 2HO· (6)

These findings were further supported by [27], who investigated direct and indirect photolysis of H_2_S emitted from wastewater treatment plants under combined 254/185 nm light in different gas matrixes (Ar, air, and O_2_) and different relative humidity values. Like Li et al. [26], this later study also reported enhanced H_2_S oxidation in humidified air. Furthermore, in the argon atmosphere, where the absence of oxygen eliminates ·OH and O_3_ formation, the H_2_S removal rate was reduced. These observations by Xu et al. [27] further support the conclusion that direct photolysis of H_2_S was less efficient than its oxidation by ·OH. Interestingly, in the O_2_ atmosphere, the H_2_S removal rate was lower than in air, which was attributed to the scavenging of the generated ·OH radicals by the generated O_3_ (present in high levels under such conditions) to form less reactive HO_2_· radicals [27]. Although these VUV studies of hydrogen-sulfide oxidation were not carried out in an aqueous solution, they still indicate the potential of this radiation for chemical-free AOP to treat H_2_S contamination in anoxic waters, utilizing in situ ·OH radicals formation following water photolysis under wavelengths <200 nm.

The present study addresses this gap and investigates the applicability of the VUV-based AOP for the removal of H_2_S both in synthetic solutions and real groundwater samples.

## 2. Results

### 2.1. Photochemistry of Sulfide in Synthetic Solutions

**Figure 1:**
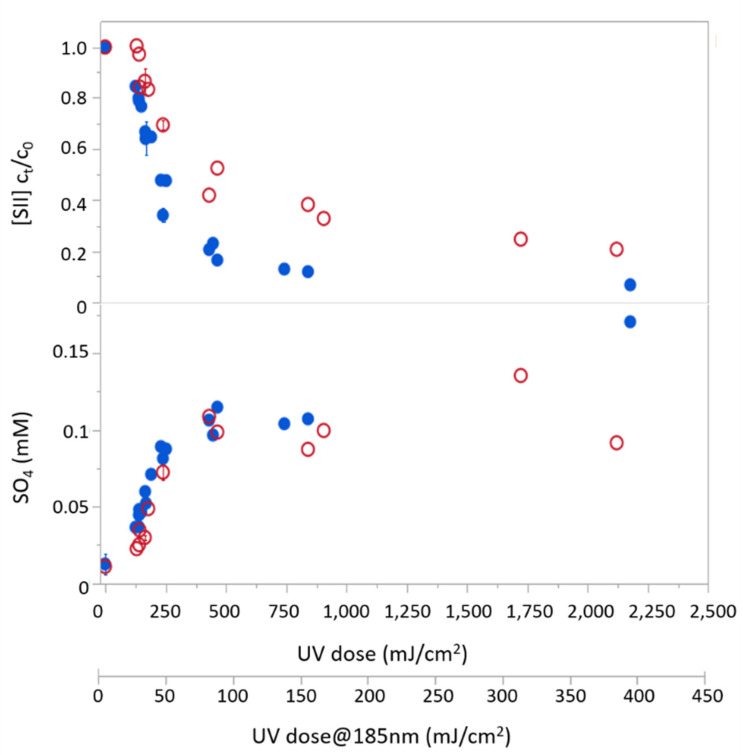
Sulfide degradation (top) and sulfate generation (bottom) using two different lamps: UV-C/VUV (254/185 nm; solid blue circles) and UV-C (254 nm; blank red circles) as a function of UV dose.

Sulfide removal was first examined in distilled water solution under the 254 nm lamp and the combined lamp, which emits at 254/185 nm. Figure 1 depicts sulfide concentrations measured in the outflow from the reactor (C_t_) normalized to its concentration at the reactor’s entrance (C_0_) as a function of radiation doses (between 0 and 2200 mJ/cm^2^) for both lamps. The observed reduction in sulfide concentration was accompanied by sulfate formation, both increasing with exposure dose. This clearly indicates photodegradation of sulfide under either 254 nm or 254/185 nm lamps (Figure 1). While both lamps have similar overall output fluence, the small addition of VUV radiation in the combined lamp spectra (with 185 nm irradiation accounting for ~14% of its total photon flux) yields a much faster degradation rate of H_2_S. At the lowest examined radiation dose (~150 mJ/cm^2^), the combined lamp yielded sulfide removal of about 25%, compared with ~3% achieved by the 254 nm lamp, and under radiation dose of about 500 mJ/cm^2^ sulfide removal under the combined lamp was almost twice as the removal obtained under 254 nm (about 80% and 45% removal, respectively).

H_2_S removal rates via direct photolysis at 254 nm and its degradation via direct and indirect photolysis under the combined lamp, followed first-order kinetics with apparent degradation rate coefficients of (0.8 ± 0.1) × 10^−2^ and (1.5 ± 0.1) × 10^−2^ s^−1^, respectively.

**Figure 2:**
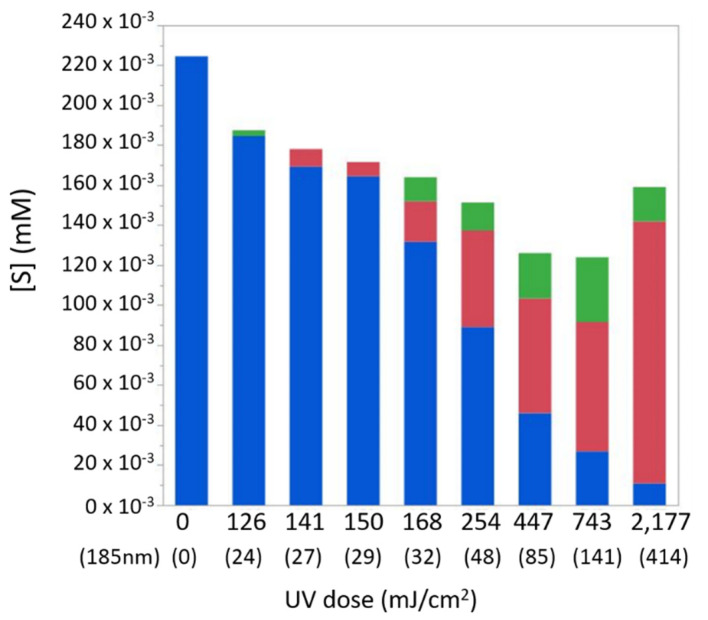
Sulfur molar balance at different radiation doses of the combined radiation (254/185 nm), based on measurements of sulfide ([SII]; blue bars), sulfate (SO_4_^2−^; red bars), and sulfite (SO_3_^2−^; green bars) concentrations. Concentrations shown for a dose of 0 mJ/cm^2^ represent the composition of reactor effluent when the lamp was off, which served as a blank.

While the sulfide–sulfate analysis shown in Figure 1 clearly indicates the photo-oxidation of H_2_S to SO_4_^2−^, looking into the results in detail shows the incomplete molar balance for sulfur. This suggests the formation of additional sulfur-containing intermediates, which are not measured by the common sulfide–sulfate methods. Indeed, additional chemical analysis revealed sulfite (SO_3_^2−^) formation along the photochemical process. Figure 2 illustrates the decrease in sulfide concentration and the increase in sulfate and sulfite concentrations with increasing radiation doses. The sulfite formed narrows the gap in the sulfur species mass-balance but not completely, suggesting the formation of additional sulfur-containing transformation products that were not measured in this study. As can be seen in Figure 2, this missing S-containing specie(s) peak around irradiation doses of 450–750 mJ/cm^2^, while at higher doses, it is further oxidizing to sulfate. As no change in the solution turbidity (using 2100P turbidimeter, HACH, Loveland, CO, USA) and particle size and number distribution (using AccuSizer^®^ FXnano, PSS, Port Richey, FL, USA; size range 0.2–200 µm) was observed upon irradiation, the formation of particulate elemental sulfur seems to be negligible.

**Figure 3:**
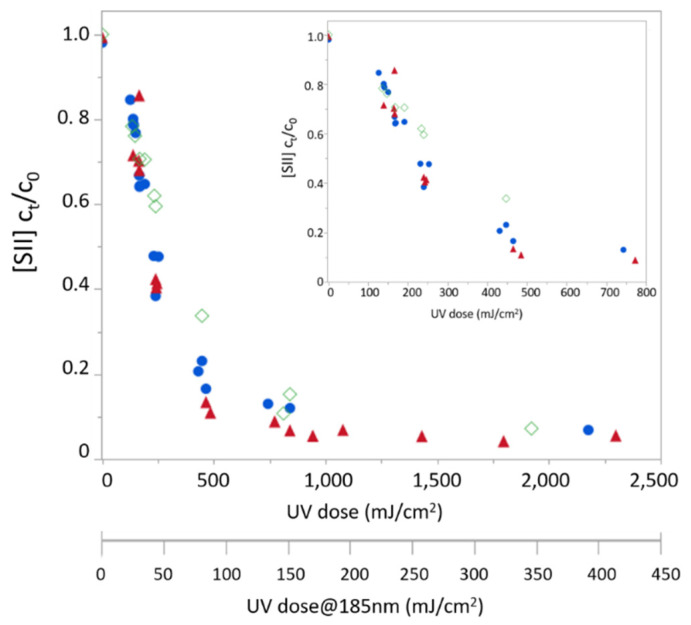
Sulfide removal [SII] under combined radiation (254/185 nm) as a function of radiation dose in distilled water solutions containing different carbonate concentrations. Insert figure zooms on UV does of range 0–800 mJ/cm^2^. Blue circles, red triangles, and green diamonds represent total carbonate concentrations of 0, 1.5, and 3 mM as CaCO_3_, respectively.

The enhanced H_2_S removal rate observed in the presence of VUV radiation can result from its oxidation by ·OH radicals generated via water photolysis under 185 nm, as well as due to its direct photolysis under 185 nm. In order to assess the relative effect of hydroxyl radicals on sulfide removal rate, photochemistry experiments were performed with distilled water with the addition of carbonate at concentrations of 0, 1.5, and 3 mM as calcium carbonate. HCO_3_^−^ and CO_3_^2−^ exhibit minor absorption at 185 nm but strong reactivity toward ·OH radicals [22]. Figure 3 shows a moderate reduction in sulfide photodegradation under increasing bicarbonate concentrations (within the range commonly found in groundwater), yielding degradation rate coefficients of (1.5 ± 0.1) × 10^−2^, (1.4 ± 0.1) × 10^−2^ and (1.0 ± 0.1) ×10^−2^ s^−1^ for distilled water solutions containing 0, 1.5, and 3 mM AS CaCO_3_, respectively. Worth noting that in a separate set of experiments, no significant difference in the H_2_S degradation rate coefficient was observed in distilled water containing 1.5, 3, and 6 mM as CaCO_3_. The rate observed under the highest tested carbonate concentration (1.0 × 10^−2^ s^−1^) is very close to that observed during direct photolysis under 254 nm alone (0.8 × 10^−2^ s^−1^). While the difference between these two rates, 0.2 × 10^−2^ s^−1^, lies within the error range of the experimental data, it can provide some upper estimation for the contribution of direct photolysis of H_2_S at 185 nm. The obtained rates suggest that under the current experimental conditions, oxidation by photogenerated ·OH radicals has a larger contribution to H_2_S enhanced degradation rate under exposure to VUV radiation. These results fall in line with previous findings for gaseous H_2_S photo-oxidation under VUV irradiation [26,27].

Despite the promising results of VUV-based AOP for H_2_S removal in synthetic solutions, in natural groundwater, the process efficiency is likely to decrease due to the presence of other species that may absorb VUV radiation (e.g., nitrate and sulfate), hence reducing the ·OH production rate following water photolysis and/or compete with the target pollutants (sulfide species) for the generated OH radicals (i.e., OH-scavengers). The following section investigates the removal of H_2_S in real groundwater.

### 2.2. Photochemistry of Sulfide in Natural Groundwater

Samples of groundwater from two boreholes, Faran and Tsofar, located in southeast Israel (Arava Valley region), were obtained from the Israel National Water Company. Chemical and physical characterization of these samples (conducted prior to the photochemical experiments) is provided in Table 1. Since the concentration of sulfides in the Faran groundwater sample was low (1.7 mg-S/L), 10 mg-S/L of sulfide were added to it before photodegradation experiments were performed. Onsite monitoring data indicate average sulfide levels in these boreholes in the range of 10–20 mg-S/L as well as dissolved oxygen concentration <0.1 mg/L (Israel National Water Company, personal communication). Hence, the chemical analysis performed in our laboratory (Table 1) suggests partial oxidation of the water samples during their transport.

**Figure 4:**
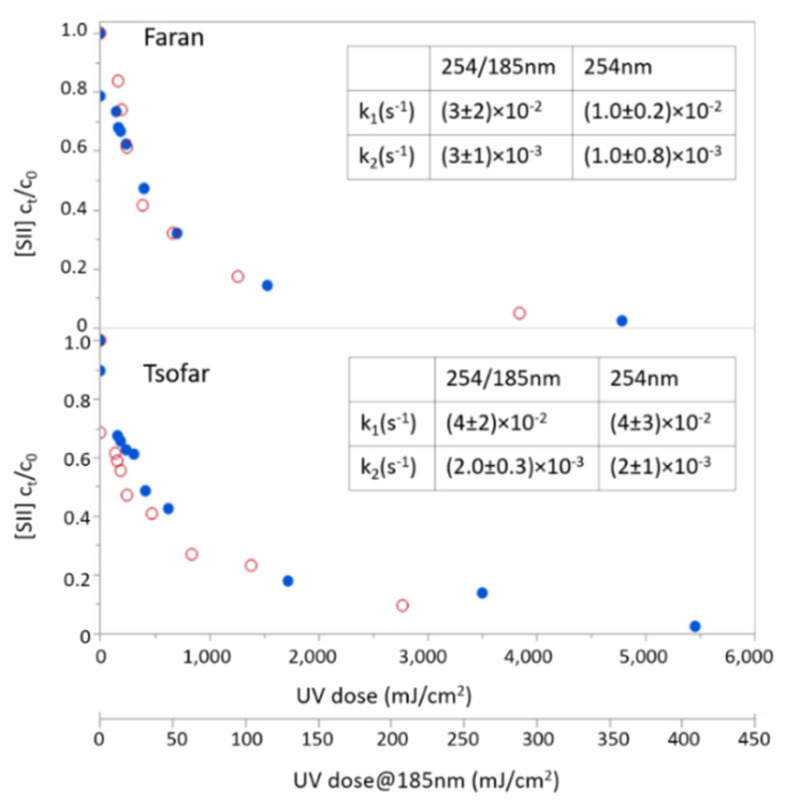
H_2_S removal from natural groundwater (normalized concentration to initial concentration) using a 254 nm lamp (blank red circles) and the combined 254/185 nm lamp (solid blue circles) in different radiation doses. Rate coefficients depicted in the inserts are the coefficients obtained from the double exponent fitting: [H_2_S] = a·exp(−k_1_ × dose) + b·exp(−k_2_ × dose).

Figure 4 depicts sulfide photodegradation rates in these two natural groundwater sources under the combined radiation (254/185 nm) and the 254 nm alone with radiation doses up to 5500 mJ/cm^2^. Sulfide was efficiently removed from the natural groundwater by the two examined lamps, with no clear preference between them, with over 95% sulfide removal being achieved at radiation doses higher than 3000 mJ/cm^2^ for both lamps (Figure 4).

Interestingly, removal kinetics of H_2_S in the natural groundwater samples showed a better fit to double exponential decay than to single exponential decay. This may suggest that in this water matrix, there are other processes in addition to oxidation by ·OH that lead to sulfide degradation at a slower rate (e.g., oxidation by secondary oxidants, such as CO_3_^2−^ and SO_4_^2−^, formed in the irradiated solution). Comparison of the rates observed in DIW and groundwater samples indicate different trends for 254 nm and the combined (lamp) 185/254 nm. Under 254 nm irradiation, removal of H_2_S in DIW was slightly less efficient than in groundwater, depicting 50% H_2_S removal at UV doses of about 550 and 400 mJ/cm^2^, respectively. An opposite trend was observed under the combined radiation, where 50% H_2_S removal was obtained at UV doses of 250 and 400 mJ/cm^2^ in DIW and groundwater, respectively. These opposite trends are likely a result of different dominant degradation pathways of H_2_S under the two irradiation conditions. Under 254 nm, sulfide removal in DIW occurs mainly via direct photolysis [12], and it is possible that in groundwater, this radiation generates secondary oxidants, for example, due to nitrate photolysis [22], that contributes to sulfide oxidation. Under the combined radiation, on the other hand, a significant portion of the sulfide loss results from its oxidation by ·OH radicals that are generated via water photolysis under VUV radiation (i.e., indirect photolysis). In such a case, the presence of other substances that are naturally present in groundwater, such as bicarbonate, sulfate, and chlorides, results in competition upon the generated radicals and a consequential reduction in the observed H_2_S oxidation rate. Worth noting that while low concentrations of sulfate can contribute to the AOP efficiency due to the formation of ·OH upon its photolysis [22], at high concentrations (as observed here), sulfate may become a significant inner VUV filter and ·OH scavenger, thus adversely affecting the process (Barki and Dubowski, unpublished data).

·OH consumption rates by the main substances present in the groundwater were compared in order to estimate their relative importance as ·OH scavengers (Table 2). The results indicate that chloride ion was the dominant scavenger, followed by bicarbonate. Chloride ions may further interfere with the AOP by acting as inner filters for VUV radiation. Nevertheless, the reaction of Cl^−^ with ·OH is reversible (especially at neutral-alkaline pH), which is expected to moderate its adverse effect on the process efficiency. Similarly, sulfate photolysis also generates sulfate radicals that can further oxidize target pollutants (a positive effect was previously observed at sulfate concentrations of up to ~50 mg/L) [22].

**Figure 5:**
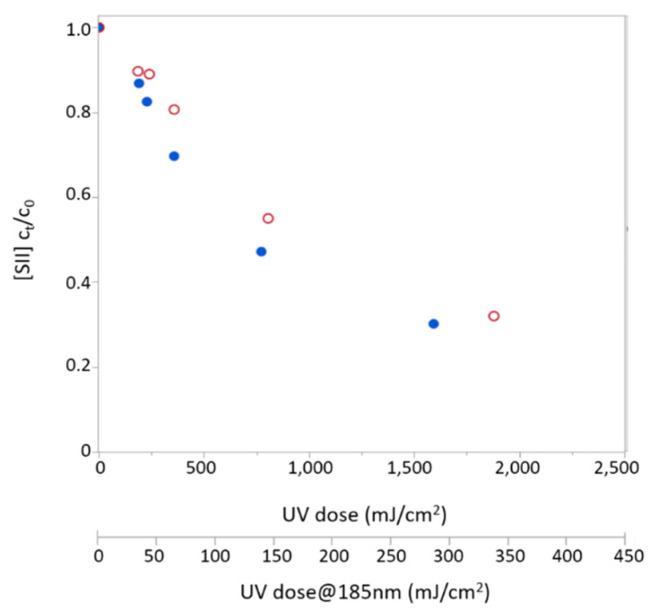
H_2_S removal from oxygen-free natural groundwater from Faran (concentrations normalized to initial concentration) using a 254 nm lamp (blank red circles) and a combined 254/185 nm lamp (solid blue circles) in different radiation doses.

Tzvi and Paz [12] showed improved removal of H_2_S from natural groundwater upon irradiation by 254 nm light with the addition of dissolved oxygen to the aqueous solution. As groundwater containing high levels of sulfide is expected to be quite anoxic with reducing conditions, it was very interesting to examine the impact of VUV addition to incident radiation on sulfide removal in such an oxygen-free solution. Hence, photodegradation experiments using the combined (254/185 nm) and 254 nm lamps were re-performed for Faran groundwater after its dissolved oxygen concentration was reduced close to zero (by adding sodium sulfite). The results of these photochemistry experiments in oxygen-free groundwater indicated some advantage for the combined lamp over the 254 nm lamp with removal rates of (5.0 ± 0.3) × 10^−3^ and (3.0 ± 0.4) × 10^−3^ s^−1^, respectively. Although the difference in the degradation rate coefficients is relatively small, it was found to be statistically significant (Figure 5).

### 2.3. Energy Demand for Photochemistry

In order to evaluate the efficiency of the sulfide removal from distilled water and from groundwater, using the 254 nm lamp and the combined lamp (254/185 nm), the energy demand for 50% removal of the initial sulfide concentration was assessed (Table 3). For H_2_S degradation in distilled water matrix (Section 2.1 above), the combined lamp was found to be significantly more energy-efficient compared to the 254 nm lamp (requiring, for example, half the energy demand of the 254 nm lamp for 50% removal of the initial H_2_S concentration). However, for natural groundwater, no clear advantage in energy requirement for the combined lamp was found. Nevertheless, when the dissolved oxygen in the groundwater was removed, as is the case in such groundwater at source (onsite), a clear preference for the combined lamp was observed (Table 3). Assuming electrical energy cost of 7 × 10^−2^ USD/kWh [35], the cost of 50% H_2_S removal under the combined lamp is estimated as 0.08, 0.17 and 0.43 USD/m^3^ for DIW, groundwater and oxygen-free groundwater, respectively. These costs are much lower than those recently reported by [36], who estimated a cost of 17–165 USD/m^3^ (depending on the extent of oxidants addition) for full removal of high concentration of sulfide ions from industrial wastewater effluent using cavitation processes. Unfortunately, we were unable to find any additional report addressing hydrogen sulfide removal from water/wastewater by AOP application. Yet, it worth noting that the obtained costs for H_2_S removal for the proposed VUV-AOP seem to fall within the range previously reported for the removal of other pollutants from groundwater using classical UV-H_2_O_2_ and UV-O_3_ AOPs ([35] and references therein).

To summarize, the current study clearly shows the potential of utilizing VUV-based AOP for treating H_2_S contamination in groundwater as a chemical-free treatment, making it especially attractive to remote small treatment facilities. However, considering the radiation requirements, it seems that it would be beneficial to add some pretreatment to reduce interferences from substances present in groundwater (e.g., bicarbonate chloride and sulfate) that compete with the sulfide for the generated ·OH radicals. For groundwater not contaminated by high levels of chloride and nitrates, bicarbonate and carbonate ions are major ·OH scavengers [20]. Hence, in this case, a simple reduction of pH prior to oxidation (converting these ions to the less reactive H_2_CO_3_) should improve the process efficiency. It should be noted that such pretreatment is likely to be needed for any AOP process that utilizes ·OH radicals for pollutants oxidation.

The additional advantage of adding VUV light to applied radiation in AOP is its ability to enhance the removal of pollutants (other than sulfide) that are insensitive to direct photolysis under conventional UV-C (254 nm) irradiation. The development of new UV irradiation sources with high fluence at the VUV range of 180–190 nm (compared to LP-Hg lamps that emit only a small percentage of their fluence at 185 nm) is highly desirable for promoting the use of VUV radiation in AOP treatments.

## 3. Materials and Methods

### 3.1. Chemicals

In total, 3.75 g of Na_2_S·9H_2_O were dissolved in 500 mL distilled water creating a standard solution and was stored at 4 °C (pH~10.2) in dark conditions. A daily solution was prepared from the standard solution at a sulfide concentration of 9–11 mg/L while keeping initial pH of 8.

For each experiment, the sulfide and sulfate concentrations were measured before and after irradiation. Sulfide was measured according to the colorimetric method [37]. The examined water sample was diluted by distilled water according to the expected sulfide concentration. A 2.3 mL water sample was inserted into a test tube containing 0.2 mL of a reagent mixture (0.5 g/L *N,N*-dimethyl-*p*-phenylenediamine + 0.75 g/L FeCl_3_·6H_2_O + HCl)). The solution was mixed for 20 min, which is the time required for full-color development, then the color is stable for many hours. Color intensity was measured at 670 nm by a spectrophotometer (Genesys 10uv scanning, Thermo Fisher Scientific, Madison, WI, USA). The colorimetric method was calibrated using the iodometric method [38].

Sulfate (SO_4_^2−^) was measured using Ion Chromatograph (881 Compact IC pro, Metrohm, Switzerland), and in addition, for closing the sulfur balance, sulfite (SO_3_^2−^) concentrations were measured by the iodometric titration method (4500- SO_3_^2−^ [38]).

### 3.2. Experimental Setup

**Figure 6:**
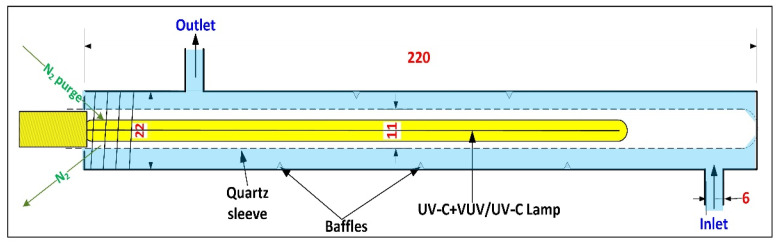
A scheme of the continuous-flow photoreactor (after [21]).

Sulfide photodegradation experiments were executed using a continuous-flow photoreactor. The reactor consists of a circular glass tube of 22 mm inner diameter and 200 mm length, with the radiation source located along its centerline (Figure 6). Two types of LP mercury lamps (input power 6.6 W, 17.8 cm long, Jelight Inc., Irvine, CA, USA) were used: a combined UV-C/VUV lamp emitting at both 254 and 185 nm wavelengths (model 81-3306-7; output intensity at the lamp surface of 30 mW/cm^2^ and 1.3 mW/cm^2^ at 254 nm and 185 nm respectively) or UV-C lamp emitting at 254 nm only (model 78-2046-7; 30 mW/cm^2^). The examined lamp was positioned inside a circular quartz sheath (11 mm inner diameter, Superasil^®^ 310, Heraeus, Germany), transparent to both wavelengths. The quartz sheath was continuously purged with N_2_ (99.999%) at a flowrate of 15 mL/min to prevent the formation of ·OH and O_3_ in the confined space between the lamp and the sheath. The width of the water film flowing around the quartz sheath was 5.5 mm. To promote mixing, baffles were added along the inner side of the reactor outer wall.

Inflow rates into the reactor varied between 100 and 400 L/min using a peristaltic pump (Cole-Parmer Ltd., Chicago, IL, USA). Average retention times in the reactor as a function of flowrate were calculated based on a tracer study using Rhodamine-A in step-input experiments [39,40].

The lamp irradiation doses at 254 nm and 185 nm as a function of the flowrate were measured with Iodide–Iodate [41] and H_2_O_2_ [42] chemical actinometry, respectively. Measured photon fluxes were, respectively, (5.2 ± 1.3) × 10^15^ and (7.2 ± 1.8) × 10^14^ photons s^−1^ cm^−2^ for the 254 nm and 185 nm.

### 3.3. Photodegradation Experiments

The efficiency of sulfide removal from the water was investigated under two lamps: LP-Hg lamp emitting at 254 and 185 nm (i.e., termed combined lamp hereafter) and LP-Hg lamp emitting only at 254 nm. Sulfide removal was examined in four different aqueous solutions: (a) Deionized water (DIW); (b) Deionized water containing Calcium carbonate (150–300 mg/L^−1^ as Calcium carbonate) that served as an ·OH scavenger; (c) Natural groundwater sampled from two boreholes located at the southern part of Israel (Arava region; Tzofar and Faran); and (d) Oxygen free natural groundwater (Tzofar and Faran). Each solution was pumped into the reactor at a different flowrate controlling the UV dose. All experiments were performed in duplicates.

## Figures and Tables

**Table 1:** Chemical and physical characterization of two natural groundwater sources from the Arava Valley (Israel).

Parameter	Tsofar	Faran
pH	7	7.03
Alkalinity (mg/L-CaCO_3_)	293	259
E.C. (µS/cm)	3760	1410
O.D. 405 (Abs./cm)	0.012	0.002
O.D. 254 (Abs./cm)	0.171	0.031
Turbidity (NTU)	21.1	3.9
H_2_S mg-S/L	10.5	1.7
SO_4_^2−^ mg-SO_4_/L	625.7	681.4
Cl mg/L	429.9	378.6
Total hardness (mg-CaCO_3_/L)	980	1000
Ca^2+^ mg/L	0	272
Mg^2+^ mg/L	238	77.8
TOC (mg/L)	1.168	0.6867
DOC (mg/L)	1.085	0.3735
Total N (mg/L)	1.85	0.6356

**Table 2:** The ·OH scavenging capacity of the main substances naturally present in groundwater.

	Concentration ^a^		k (·OH)	·OH Scavenging	ε_185nm_	extinction (185 nm)
	mg/L	M	s^−1^ M^−1^	s^−1^	M^−1^ cm^−1^	cm^−1^
HCO_3_^−^	259–293	(4.5–4.8) × 10^−3^	8.50 × 10^6^ [29,30,31]	(3.6–4.1) × 10^4^	269 [22]	1.14–1.29
Cl^−^	378.6–429.9	(1.1–1.2) × 10^−2^	3.00 × 10^9^ [32,33]	(3.2–6.3) × 10^7^	3063 [34]	32.67–37.1
SO_4_^2−^	681.4–625.7	(6.5–7.1) × 10^−3^	-	-	146 [34]	0.95–1.04
WATER		55.4	-	-	0.029	1.60 [34]
DOC	0.3735–1.085	(3.1–9.0) × 10^−5^	6.60 × 10^8^ [31]	(2.1–5.9) × 10^4^	1402 [34]	0.04–0.13

^a^ Concentrations of major ions are based on the natural groundwater analyzed (Faran and Tsofar, Table 1).

**Table 3:** The UV dose required for 50% sulfide removal and energy demand for 50% removal in different photodegradation experiments.

Sulfide Dissolved in	Lamp Type	UV Dose Required for 50% Removal at 254 nm Wavelengths (mJ/cm^2^)	UV Dose Required for 50% Removal at 185 nm Wavelengths (mJ/cm^2^)	Energy Demand for 50% Removal (kWh/m^3^)
DIW	254/185	238 (±12)	45 (±1.7)	1.2
DIW	254	417 (±36)		3.0
Faran	254/185	317 (±20)	70 (±2.8)	2.4
Faran	254	286 (±12)		2.4
Faran-oxygen free	254/185	690 (±14)	131 (±2.0)	6.1
Faran-oxygen free	254	1045 (±72)		11
Tsofar	254/185	398 (±34)	77 (±4.8)	2.7
Tsofar	254	248 (±27)		2.3

## Data Availability

Not applicable.

## Sample Availability

Samples of the compounds are not available from the authors.
